# Supplementary material for: Tensiomyographical responsiveness to peripheral fatigue in quadriceps femoris
Source: PeerJ. 2020 Feb 28;8:e8674. doi: 10.7717/peerj.8674 (PMC7050546; doi:10.7717/peerj.8674)
Supplement: Supplemental Information 1 [file peerj-08-8674-s001.docx]

Variable gender:

1- Male

2-Female
